# From Micromeritics to Nanomeritics: A Review and Integrative Framework for Bridging the Physicochemical-Biological Continuum

**DOI:** 10.3390/pharmaceutics18101222

**Published:** 2026-09-26

**Authors:** Natasha Amaravadi, Benjamin M. Yee, Patrick J. Sinko

**Affiliations:** Department of Pharmaceutics, Ernest Mario School of Pharmacy, Rutgers, The State University of New Jersey, Piscataway, NJ 08854, USA; natasha.amaravadi@rutgers.edu (N.A.); bmy20@pharmacy.rutgers.edu (B.M.Y.)

**Keywords:** pharmaceutical micromeritics, nanomeritics, nanoparticles, particle size distribution, polydispersity index

## Abstract

Few nanoparticle-enabled medicinal products (NEPs) are commercially available today, despite the introduction of Doxil, the first Food and Drug Administration (FDA)-approved nanotechnology drug product, in 1995. One hurdle in developing NEPs is the need for specialized tools to characterize and detect nanoscale materials under various conditions (e.g., during manufacturing or after administration into the body). Another significant scientific challenge lies in characterizing NEPs. For example, interpreting particle-sizing results is difficult because many conventional detection methods do not allow scientists to analyze or confirm the size and properties of NEPs. While characterization methods for larger particles (1 µm to 1 mm) are well established, the same cannot be said for nanoscale particles. In fact, the physical and biopharmaceutical properties of particles made from the same material but differing in size can vary significantly, particularly for particles measuring approximately 1 nm to 100 nm. These challenges have led to a re-evaluation of the characterization and interpretation tools essential for developing NEPs. In this review, pharmaceutical micromeritics is first summarized, including the characterization techniques for particles in the 1 µm to 1 mm size range. Then, the concept of nanomeritics is discussed. Nanomeritics differs from micromeritics by focusing on the characterization of nanoscale particles and extends the field to its implications in biological environments. Nanomeritics is introduced by (1) defining the nanoparticle size range, (2) providing a comprehensive overview of the methods to characterize nanoparticles, (3) proposing orthogonal characterization guidelines, and (4) identifying how NEPs interact in biological environments. In this review, the methodologies and limitations of particle sizing and characterization methods are also discussed.

## 1. Introduction

J. M. DallaValle, widely regarded as the father of micromeritics, defined it as the science of small particles in his 1943 book, “Micromeritics—The Technology of Fine Particles” [1]. He derived his new term from the Greek words for “small” (mikros or μικρός) and “part” (meros or μέρος). Micromeritics is used in a vast range of fields, including food science [2], concrete [3], rocket propulsion [4], and powder metallurgy [5]. The pharmaceutical sciences are no exception, as particles have been the building blocks of medicinal formulations for over one hundred years, and particle properties are essential to the performance and stability of pharmaceutical formulations [6,7,8,9]. DallaValle defined the micromeritic particle size range as 0.1 µm (100 nm) to 105 µm, and he compiled widely scattered information on particle measurement, size distribution, and packing arrangements, creating a guide to standardize the characterization of small particles based on their behavior and characteristics. While micromeritics has been extensively used in the pharmaceutical sciences [10], its application does not extend to very small particles, i.e., nanoscale particles with diameters less than 100 nm. Additionally, some micromeritic properties do not apply to nanoscale particles (e.g., the Hausner ratio). Nanoscale particles lack robust characterization approaches and protocols needed to develop successful pharmaceutical products [11].

The term nanomeritics, first introduced in 2024, refers to the study and characterization of pharmaceutical particles and structures in the size range of ~5 nm to ~1000 nm [12]. Although this range may be refined further in the future, this size range was justified based on the current use of the terms relating to “nano” in the literature [13]. For example, gold nanoparticles (~5 nm) are among the smallest reported nanoscale drug delivery systems, but the literature often refers to pharmaceutical nanotechnology drug delivery systems at the upper end of the size range (i.e., nearing 1000 nm) [12,13,14]. There have been efforts to lay the groundwork for a comprehensive strategy for nanoscale particle characterization, given the unique complexities posed by nanoparticle-enabled medicinal products (NEPs) (e.g., chemical composition, average particle size, polydispersity, dispersion stability, among others) compared with “classical” small-molecule drugs [11]. Understanding the properties of NEPs is critical for describing their behavior across various systems and for determining the safety and efficacy of particles in medicinal products [12]. Nanomeritics elucidates the complex and unique requirements for the proper characterization of NEPs. This review will discuss micromeritics, introduce nanomeritics, and highlight key areas of distinction and need.

Micromeritics encompasses a vast range of particle sizes (Figure 1), each size with its own advantages. For example, some of the largest pharmaceutical particles include coarse powders (~3 mm) and granules (~100–500 µm) [15]. To put it in perspective, coarse powder particles are about 100 times larger than a human skin cell [16]. Micron-sized drug carriers, such as microspheres, are comparable in size to lymphocytes (8 μm) and can be engulfed by macrophages (2 μm) [17,18,19]. Finally, nanoparticles, ideally less than 30 nm, can diffuse through the vasculature in both normal and tumor microenvironments, while those below the kidney filtration threshold (~8 nm) are subject to renal clearance [20,21,22,23,24,25].

While pharmaceutical micromeritics technically spans 1 nm to a few millimeters, micromeritic properties alone do not adequately characterize nanoparticles on the smaller end of this range. For example, Danaei et al. established that lipid-based nanocarriers, such as nanoliposomes, which typically range from 50 to 150 nm, can accumulate in tumor tissues due to leaky vasculature-induced enhanced permeability and retention (EPR) effect and minimize clearance by the mononuclear phagocyte system (MPS) [27]. Danaei et al. report that reducing particle diameter to 50–80 nm can decrease phagocytic uptake and extend circulation time in vivo. Micromeritics characterizes the mechanical properties of particles (e.g., elasticity and stiffness) and non-biological interactions [26]. For this reason, micromeritics cannot adequately characterize how the average particle size of lipid-based nanocarriers affects biological parameters, such as phagocytic uptake and circulation time. Alternatively, nanomeritics examines how properties such as particle size, chemical composition, polydispersity, and dispersion stability influence biological interactions. Studying nanoparticle-specific properties is critical for advancing NEPs; accordingly, the concept of nanomeritics is introduced in this review.

Micromeritics evaluates powder mechanics based on particle mass and gravitational interactions. In contrast, nanoscale behavior is governed by interfacial surface forces, emphasizing the importance of surface phenomena. The operational boundary of a nanoparticle is dynamic, varying continuously with environmental factors such as ionic strength, pH, solvent polarizability, and protein concentration, highlighting the dynamic behavior of nanomaterials. There are several key limitations of micromeritics at the nanoscale. For example, at the microscale, sizing relies on methods that assume well-defined spherical equivalent diameters. However, at the nanoscale, Rayleigh scattering dominates, making it exceptionally difficult to detect nanoparticles in the presence of even trace amounts of aggregates [28]. This is because a small increase in particle size causes a massive increase in scattered light. To overcome this limitation of scattering methods (e.g., Dynamic Light Scattering, or DLS), orthogonal single-particle methods such as Field-Flow Fractionation (FFF) or Nanoparticle Tracking Analysis (NTA) can be used (discussed later in this review). Since DLS measures a hydrodynamic diameter, inclusive of the solvation shell and surface-adsorbed layers, rather than the physical core boundary defined in micromeritics, orthogonal methods should be used to confirm particle size. In micromeritics, material properties such as density are assumed to be constant regardless of particle size. In direct contrast, at the nanoscale, bulk physical properties break down [29,30,31,32]. Therefore, classical micromeritic models that input bulk material constants yield systemic errors when applied to nanomaterials. At the microscale, cohesive forces (e.g., van der Waals and electrostatic) are present but can often be overcome. However, at the nanoscale, interparticle cohesive forces, specifically van der Waals, capillary, and electrostatic interactions, outweigh gravitational forces by orders of magnitude. Standard micrometric metrics (e.g., Hausner Ratio) are widely used to assess powder cohesion and flowability. However, at the nanoscale, these classical flow indicators become meaningless. Bulk and tapped density measurements reflect the fractal packing geometry of aggregate networks rather than the packing behavior of individual primary particles [33,34,35]. This environmental sensitivity influences properties such as particle size (discussed later in this review). Compared to the microscale, the surface area per unit mass increases exponentially, emphasizing the importance of surface phenomena that drive non-equilibrium processes such as Ostwald Ripening.

## 2. Micromeritics

By applying measurable attributes to microscopic particles, the macroscopic behavior of pharmaceutical systems can be better understood. Micromeritics focuses on the holistic characterization of particles, which requires an understanding of their properties, including size, shape, surface (area, charge, mechanical characteristics, and microstructure), with properties specific to powders (e.g., flowability, density, and compressibility) also needing consideration [1,30,36,37,38]. While micromeritics spans multiple disciplines, for the purpose of this article, we will focus on its application to the pharmaceutical sciences [3,4,5,39,40,41,42]. Size has significant implications for particle behavior as it travels from the manufacturing belt through the human body, directly affecting powder flow and packing, sedimentation, formulation stability, and dissolution rate [37,43,44,45,46]. For example, efforts have been made to control particle size during manufacturing to tune agglomeration and growth, producing larger, free-flowing particles (>250 µm) better suited for granulation and tableting, rather than promoting breakage and nucleation of smaller particles (>10 µm) [47]. However, the intricacies of what particle size means should be examined first. Aside from gas bubbles and emulsions, particles do not naturally occur as perfect spheres and, as such, cannot be described by a singular dimension. The concept of equivalent spheres is applied to irregularly shaped (i.e., asymmetric) particles and relates them to the diameter of a hypothetical sphere with the same maximum parameter (Figure 2), which varies across models and particle-sizing instruments [48]. Simplifying the measurement of singular particles is useful, but the ultimate goal of these sizing techniques is to characterize particle populations. Often, an unprocessed sample consists of particles of different sizes, or a polydisperse sample [49,50,51,52]. The degree of non-uniformity of a sample is represented by the polydispersity index (PDI), which quantifies the particle size distribution (PSD) obtained from analytical techniques such as DLS, with 0.0 indicating perfect uniformity and 1.0 indicating full heterogeneity [27,53]. PDI and PSD will be further discussed in the following sections. Almost all real-world samples exist as a distribution of particle sizes, so it is essential to understand that a single number cannot describe the distribution of the sample. When reporting particle size distribution, different weighting parameters (volume, number, intensity) are used, and these vary across sizing techniques and software. D-values (D- or d-) are the most commonly used metrics for describing particle size distributions [26]. Percentile parameters follow the notation DxY, in which “D” denotes that a diameter is being reported, “x” specifies the weighting of the distribution (“n” corresponds to number, “v” to volume, and “i” to intensity), and “Y” represents the cumulative percentage of the sample whose particle size falls below the reported value. D-values represent the intercept of the cumulative mass curves. In contrast to D-values, which are summary statistics, the PSD represents the full particle size distribution, allowing D10, D50, and D90 to be extracted while also describing the distribution’s overall shape and breadth.

Shape contributes similarly to the bulk flowability, packing properties, and the dissolution of pharmaceutical dosage forms, and is intrinsically linked to surface area, another major determinant of physicochemical behaviors [55,56,57,58,59,60,61,62,63]. Because particles exist as complex three-dimensional objects, their description must be simplified to facilitate practical measurement and data interpretation. Among the most frequently used shape descriptors are aspect ratio, roundness, sphericity, and roughness. Aspect ratio is a dimensionless quantity that reflects a departure from a perfectly round form. While the early literature defined it as the simple ratio of length to width, the current convention, as codified in ISO 9276, defines it as the ratio of the Feret minimum length to the Feret maximum length, with values constrained to the range 0 < AR ≤ 1, where a value of 1 indicates a perfect sphere [64]. Roundness characterizes the curvature of a particle’s corners and is calculated as 4(area)/(π × LengthFeMax^2^), approaching 1 as the particle tends toward a circular profile. Roughness, by contrast, reflects fine-scale surface texture at the particle corners, specifically the jaggedness of the particle edges. As surface concavities increase, the convex perimeter grows, and the roughness value falls; this parameter is particularly sensitive to small-scale surface irregularities. Sphericity is defined as [4π(Area)]/Perimeter^2^, equivalently expressed as the square of the ratio of the perimeter of an equivalent circle to the actual particle perimeter. Like roundness, sphericity approaches 1 for near-perfect spheres, but it is comparatively more sensitive to minor surface features that inflate the measured perimeter. Imaging-based techniques such as scanning/transmission electron microscopy (SEM/TEM) are the predominant approach for shape characterization, in which a two-dimensional projection of the particle is captured, and geometric parameters are extracted through straightforward mathematical operations. Raw measurements, such as area, perimeter, and various linear dimensions, are derived directly from the image’s pixel map (Figure 3).

Surface properties (surface area, surface roughness, and porosity) exert a significant influence across nearly every stage of pharmaceutical development and manufacturing, including purification, processing, blending, tableting, and packaging, as well as downstream performance metrics such as shelf life, dissolution rate, and bioavailability [65,66,67,68]. For example, imperfect tablets can result from powder sticking to the punch surface due to the increased surface area of the powder particles [69]. In fact, the greater the surface roughness, the greater the potential for attractive forces (van der Waals, London dispersion, or hydrogen bonding) to dictate particle assembly and stability/instability in the case of coagulation and aggregation [70,71]. The importance of surface interactions is not limited to particles in solution; the density of surface ligands on engineered particles can influence their avidity for cell receptors in biological systems [72]. An increased number of surface ligands is also favorable for particle internalization into cells via receptor-mediated endocytosis, as it facilitates receptor binding and internalization [73]. Increases in porosity are also intrinsically linked to increases in surface area, thereby influencing the packing and assembly of colloids and colloidal microstructures [74,75]. When applying surface area to regularly shaped (spherical) particles, an equivalent sphere can be used; for irregular particles (rods and needles), a cylinder is more appropriate.

Just as interparticle interactions are important for physical stability, the behavior of charged particles in a dispersion medium is governed by a separate phenomenon. When immersed in a solvent, charged particles attract counterions of opposite charge that electrostatically adhere to the particle surface, forming the first of an electrical double layer known as the Stern layer. The outer or diffuse layer forms the second portion of the double layer, creating a shell of oppositely charged ions around the Stern layer [76,77]. A theoretical plane, called the slipping plane, forms the interface between the diffuse layer and the dispersion medium, at which mobile ions exist and move independently of the stationary bulk fluid [78]. The potential at this point is called the zeta potential, as reviewed by Bhattacharjee, Park, and Seo, and can be used to explain dispersion, aggregation, or flocculation [2,27,79,80,81].

The importance of mechanical properties (elasticity, stiffness, rigidity, hardness, and strength) continues to gain traction for their impact on the biological action of pharmaceutical particles. [82,83]. Manipulation of a particle’s mechanical properties can enable targeted delivery and facilitate cellular internalization and biodistribution. Tuneable hydrogels, formed from a network of linked hydrophilic polymers, exhibit a porous structure consisting mostly of water that can be easily manipulated by changes in the fluid content [84]. Forgham et al. review the mechanobiological forces and how controlling properties such as stiffness, rigidity, and elasticity can significantly affect key pharmacokinetic parameters, including bioavailability and clearance [85]. At the nanometer scale, Atomic Force Microscopy (AFM) can determine the surface topology. Compressive forces are applied to the sample to induce deformation, and the resulting indentation force–displacement curve is recorded [86,87,88,89,90]. Zhai et al. provide an in-depth review of biomechanical parameters and the suitable techniques for characterization [91]. At the micromeritic scale, mechanical properties for bulk pharmaceutical powders or ocular and topical gels can be measured via rheology through the conversion of the shear modulus to the Young’s modulus [92]. As particles progress to the nanomeritic size range, an understanding of biomechanical properties can better inform their delivery and overall efficacy, clearing a hurdle that many nanoformulations face for clinical approval.

### Micromeritic Particle Classes

Micromeritics focuses on micron-sized particles to evaluate their performance and processing in pharmaceutical applications. Pharmaceutical micromeritics applies to colloids and nano drug delivery systems (nano DDS), coarse dispersions, and powders (Figure 1) [26]. The smallest end of micromeritics includes colloids and nano DDS. Colloids are 1 nm to 500 nm in size. Nano DDS typically range from 1 to 100 nm, but larger nano DDS can exist [84,93,94,95,96]. A colloid is a homogeneous noncrystalline substance in which insoluble particles are dispersed in a medium. The particles undergo Brownian motion and remain suspended in the dispersion medium. Colloidal stability is assessed by measuring charge properties through zeta potential [2,81]. In biological environments, the interface between colloidal particles and their dispersion medium affects adsorption (i.e., particles sticking to biological surfaces) and capillarity (the tendency to move due to attractive forces) [12,97,98]. For example, human blood contains colloidal dispersions of serum albumin (>1 nm in size), and its high binding affinity can enhance drug binding [12,99,100,101]. Furthermore, specific particle properties, such as particle size, shape, and surface charge, strongly influence the behavior of colloidal dispersions in nano DDS. Fullstone et al. studied the Brownian motion of nanoparticles as they pass through blood vessel walls and concluded that thermal fluctuations, combined with nanoparticle properties such as size and polydispersity, affect laminar flow through blood vessels [20]. This example illustrates how particle properties in the smallest size range (colloids and nano DDS) influence their behavior.

The second largest group of particles within the pharmaceutical micromeritics classification is emulsions and suspensions, which typically fall within the ranges of 1–10 µm and 10–100 µm, respectively (note that different sources may categorize these ranges slightly differently) (Figure 1). An emulsion is a dispersion of one liquid into a second, immiscible liquid. Three main categories of emulsions are generally recognized in the literature: nanoemulsions (20–500 nm), microemulsions (10–300 nm), and macroemulsions (1–100 µm) [12,94,102,103,104,105]. Although nanoemulsions and microemulsions exhibit overlapping size ranges, they are distinguished primarily by their thermodynamic properties and formation mechanisms rather than just size alone. Microemulsions are thermodynamically stable, spontaneously forming systems with very low interfacial tension, whereas nanoemulsions are kinetically stable dispersions that require external energy input during preparation. Suspensions, in contrast, are solid dispersed systems in which solid particles are distributed within a liquid or semisolid phase [106,107]. Liquid suspensions may undergo flocculation, the reversible aggregation of particles that can be readily resuspended, or coagulation, the irreversible aggregation in which particles form a dense sediment [108]. Both emulsions and suspensions can be formulated at the nanoscale, in which case they fall within the colloidal size range [12]. Nanosuspensions represent a specialized class of suspensions with enhanced stability, dispersibility, and bioavailability because the smaller particles (0.1–1 µm) exhibit increased Brownian motion [12,109,110,111].

Powders and granules represent the largest particle size class within pharmaceutical micromeritics and are typically greater than 100 µm (Figure 1). Powders are a type of pharmaceutical particle that, when agglomerated, form granules. Granules are formed through granulation, in which primary powder particles adhere to form larger aggregates. There are two methods of granulation: wet granulation and dry granulation [15]. Wet granulation involves pre-blending wet binders with the active pharmaceutical ingredient (API) to form granules, which are then dried and subsequently compressed into tablets. This method ensures a homogeneous distribution of the API throughout the blend, thereby enhancing flow and compatibility. Dry granulation, however, involves blending the API and intragranular excipients without using a liquid to form ribbons, which are then milled into granules [15]. The primary purpose of granulation is to prevent segregation, improve flow, and improve compaction of the tablet dosage form [15,112,113]. Granulation is important because it ensures the proper formation of bonds between powder particles, keeping them together and preventing breakdown [114]. There are five primary particle-bonding mechanisms. The first are adhesive and cohesive forces between a thin, immobile liquid film and the primary powder particles. The thin films decrease the interparticle distance and increase the particle-particle contact area. The second mechanism involves interfacial forces between particles in mobile liquid films. The interfacial forces are crucial for forming strong bonds and solid bridges between particles [112]. The third mechanism is the primary mechanism in wet granulation, involving the formation of solid bridges between particles following solvent evaporation. The fourth mechanism involves the attractive forces, such as Van der Waals and electrostatic forces, between the solid particles. The last mechanism involves mechanical interlocking between particles, often observed in fibrous or flat particles. Together, these mechanisms bind powder particles into granules, which are ultimately used in solid dosage forms. Colloids, suspensions and emulsions, and powders and granules, comprise the size range of pharmaceutical micromeritics and are defined by a variety of particle properties that can be measured as described in the next section.

## 3. Particle Sizing

Micromeritic studies are widely used to characterize powder flow behavior and granule compressibility, thereby optimizing particle size distributions. For example, controlling particle size and distribution is critical for dry powder inhalers, where monomodal populations within the respirable range (less than a few microns) improve lung deposition and dose uniformity [115,116]. Accordingly, numerous analytical techniques have been developed to characterize particle size and morphology across the micromeritic range.

Particle characterization techniques most commonly include light scattering methods, such as DLS, and imaging techniques, including transmission electron microscopy (TEM). However, many additional techniques, such as atomic force microscopy (AFM) and laser diffraction, can also be used to characterize particles. Common particle sizing techniques and the size ranges they cover are shown in Figure 4. While size has a profound effect on particle behavior, shape also contributes significantly, particularly to surface properties such as surface area [117]. Laser diffraction is a staple technique in pharmaceutical micromeritics, characterizing particle shape and size distributions. Using multiple techniques to characterize particles can provide a deep understanding of their features.

**Figure 4:**
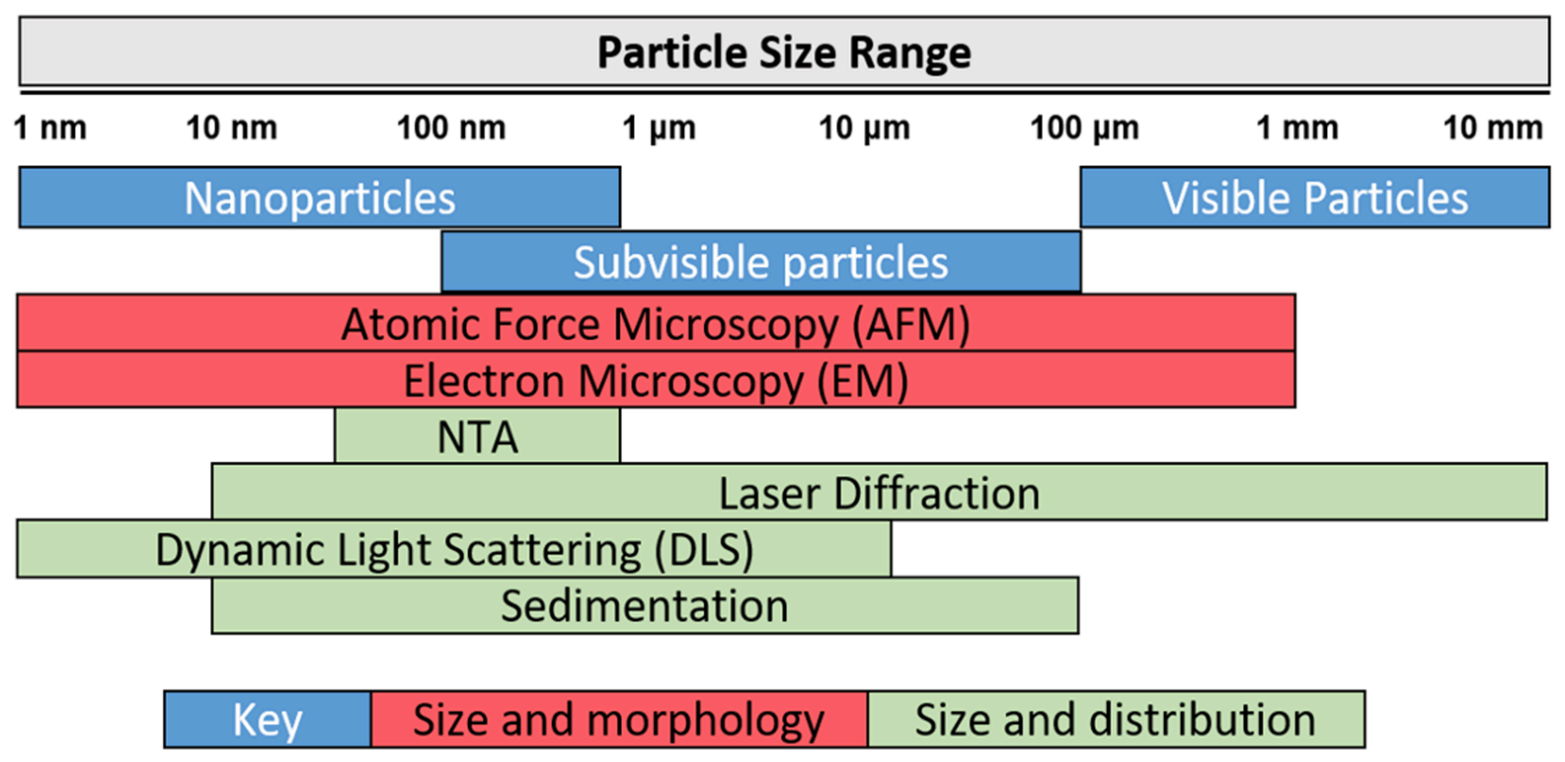
Valid size ranges of commonly used particle sizing techniques. Particle sizing techniques and the size range and applications they cover. Particle sizing techniques can be categorized as (1) imaging techniques that provide information on size and morphology (red) and (2) techniques that indicate particle size and distribution (green). Techniques are size-dependent, but there is significant overlap in the size ranges of the various techniques, spanning from nanoparticles to subvisible particles to visible particles. (Re-drawn and modified from Figure 19-7 of Martin, Pharmaceutical Micromeritics. 19-7 (2023) [26]).

### 3.1. Particle Size and Distribution

Monodisperse particle systems have a uniform size distribution, meaning they are all essentially the same size. However, most pharmaceutical particle samples are considered polydisperse, i.e., they exhibit a size distribution that varies. Because of this, it is essential to understand the shape and surface area of individual particles, as well as the particle size range, particle number, particle weight, or particle volume. Only when particles are perfect spheres, as in gas bubbles or emulsions, can they be described by a single dimension. As such, it becomes increasingly difficult to express size in terms of diameter as the irregularity of particle shape increases and the degree of asymmetry also increases [26].

When the primary property of interest is particle size distribution, measurements typically yield an intensity distribution that shows how much light is scattered across different size ranges. From this distribution, the mean particle size, standard deviation, and relative polydispersity (calculated as the standard deviation divided by the mean) can be determined directly. The overall polydispersity is converted into an overall polydispersity index (PDI), which is the square of the light scattering polydispersity. The PDI is dimensionless, and values range from 0 to 1. For a perfectly uniform sample, the PDI would be 0.0. International standards organizations (ISOs) have established that PDI values < 0.05 are more common in monodisperse samples (e.g., reference standards), while values > 0.7 indicate a broad size (e.g., polydisperse) distribution of particles (ISO standards ISO 22412:2017) [27,118].

### 3.2. Centrifugation-Based Techniques

Sedimentation is a well-established method to generate PSDs of a sample of particles by applying Stokes’ law to measure the rate of particle settling in a fluid of known viscosity [119,120,121,122]. Here, centrifugal or gravitational forces are used to separate particles, with gravitational sedimentation applicable only to larger particles (nanoparticles less than ~100 nm do not settle by gravity). Two methods, Differential Centrifugal Sedimentation (DCS) and Analytical Ultracentrifugation (AUC), separate particles based on mass, which is determined by their density and size, with resolving powers of 10 nm to 50 µm and down to ~1 nm, respectively [123,124]. Separation by DCS occurs as particles settle through a density gradient within a clear rotating disk [11,125]. As particles move outward to the edge of the disk, they alter the scattering of a monochromatic light source. These time-dependent scattering changes are recorded and converted into an intensity-based particle size distribution. In contrast, AUC exposes a clear quartz or Safire cell to centrifugal forces, generating a radial concentration profile of the sample. This profile is continuously scanned with detectors such as absorbance or interference optics to obtain a distribution c(r,t), which is then modeled to calculate sedimentation-coefficient distributions, c(s), and converted into mass-based particle-size distributions [126,127,128,129]. A key difference between DCS and AUC is in their operating speeds and, therefore, their resolving power. DCS operates at lower rotational speeds, thereby limiting its detection to particles larger than ~5 nm in dense materials such as metals and to particles larger than ~20 nm in less dense materials such as silica, polymers, or liposomes [130,131,132]. AUC, capable of producing forces above 100,000 g, can resolve much smaller species, including proteins, polymers, small molecules, and nanoparticles, down to ~1 nm resolution [11,133,134].

### 3.3. Electron Microscopy

Electron microscopy is commonly used in micromeritic studies to characterize particle morphology and can augment the characterization of nanoparticles. Transmission electron microscopy (TEM) focuses a high-energy electron beam onto a sample. Collisions between the fired electrons and those in the sample result in electrostatic interactions and the scattering of sample electrons, which are detected and used to build a 2D projection from the 3D sample [135,136]. As electrons pass through, they are scattered by internal structures such as atoms or crystalline planes. Then, the transmitted and diffracted electrons are collected and magnified by electromagnetic lenses to form an image or diffraction pattern, revealing the internal ultrastructure at atomic-scale resolution. The accuracy with which TEM measures particle composition down to the low-nanometer range, as well as its ability to provide direct images of the sample, are reasons it has become the most popular imaging technique for determining particle morphology [137].

Scanning electron microscopy (SEM) uses an electron beam to scan the surface of nanoparticles, which emits secondary electrons that form an image. These electrons interact with atoms near the surface, producing secondary electrons, backscattered electrons, and characteristic X-rays, and produce signals. The detected signals are converted into high-resolution images that characterize surface morphology, texture, and composition. SEM samples can be bulkier than TEM samples, but SEM is still considered lower resolution. SEM examines larger areas (up to several hundred square centimeters in most microscopes), generally requires moderate preparation time, and is more affordable for research and industrial laboratories [138,139].

TEM and SEM have different benefits. For example, Eaton et al. compared TEM and SEM measurements of metallic nanoparticles and observed that, under TEM conditions without shadowing or staining, almost all the signal originated from the metallic core, thereby ignoring the organic capping layer [139]. However, they found that SEM was slightly less sensitive, as sensitivity is greater for higher-atomic-number materials (this depends somewhat on the type of detector used), but that organic material may also be detected [139,140]. They measured the size of gold nanoparticles via TEM and SEM; although the mean sizes were 13.7 nm and 12.1 nm, respectively, and not significantly different, SEM produced a much wider size range and was noisier. When discerning sizes in bi-modal gold nanoparticle populations of 15 nm and 60 nm, TEM also distinguished sizes within each population more accurately than SEM.

One existing challenge in particle characterization via electron microscopy is the difficulty of imaging nanoparticles in liquid samples due to the high-vacuum requirements. Yang et al. developed a graphene-based wet-cell device that enabled in situ SEM observation of gold nanoparticles suspended in liquid, capturing their Brownian motion in real time with <5 nm resolution [140]. By using a thin, conductive graphene membrane as a window, the setup allowed SEM electrons to pass through while sealing the liquid, enabling high-resolution imaging inside the microscope’s vacuum that would otherwise be impossible with TEM. These examples illustrate the nuances in using various microscopy techniques, such as TEM and SEM, in particle characterization.

### 3.4. DLS

DLS, also known as Photon Correlation Spectroscopy (PCS) or Quasi-Elastic Light Scattering (QELS), is a well-established technique for measuring the size of particles and macromolecules in the submicron range, down to 1 nanometer or less. Published studies dating back to the mid-to-late 1990s have used DLS as the primary sizing technique for nanoparticles [141,142,143,144,145]. Batch-mode DLS is a low-resolution technique that has become a mainstay in particle sizing. DLS has significant advantages, including the need for only a small sample size, rapid and inexpensive analysis, high throughput, the ability to use non-specialized personnel, and non-invasiveness, which allows for complete sample recovery. It can also account for “soft coatings” on the particle surface, formed by stabilizers such as poly(ethylene glycol) during particle sizing.

DLS depends on thermally induced collisions between suspended particles and solvent molecules, as well as the resulting Brownian motion. When particles are illuminated with a laser, the intensity of the scattered light fluctuates over very short timescales at a rate that depends on particle size. Smaller particles are displaced further by the solvent molecules and move more rapidly. Analysis of these intensity fluctuations yields the velocity of the Brownian motion. The signal is processed through a correlator algorithm. The particle size is reported as the hydrodynamic diameter, calculated from the Stokes–Einstein relationship using the intensity autocorrelation function (correlogram) [2,11,146]. Correlograms indicate the hydrodynamic diameter and the sample’s polydispersity, providing insight into the contributions of large particles. However, the algorithm assumes a spherical particle shape and a known viscosity of the dispersion medium, neither of which is always the case and which could lead to inaccuracies in measurements if the algorithm is not properly understood.

DLS is an “ensemble” technique, meaning the diameter obtained corresponds to a sphere with the same translational diffusion coefficient as the particle being measured. The translational diffusion coefficient depends not only on the size of the particle’s core but also on any surface structure, as well as the concentration and type of ions in the medium. This means that the size will be larger than that measured by other techniques, such as electron microscopy, which removes the particle from its native environment.

DLS is not without caveats: resolving power is sacrificed for speed, making it susceptible to skewed results, especially in batch mode. Batch-mode DLS is considered a low-resolution method because the scattering intensity depends on particle size to the sixth power. The resolution is generally low since the particles are neither counted nor physically separated. While effective for monodisperse systems or samples with particles having similar size, DLS has difficulty resolving polydisperse systems [147,148]. This is because smaller particles are often masked by the higher scattering intensity of larger particles or aggregates. DLS cannot distinguish between a large particle and an aggregate of similar size and, as a result, cannot resolve the particle size distribution when plasma proteins in a biological sample interfere [149]. The raw data are analyzed mathematically using the algorithm to produce size-distribution information. However, information can be lost because the signals are often complex, nonlinear functions of size. DLS is often unable to distinguish between the PSDs of polydisperse samples comprising two or three particle populations within similar size ranges [124,146,147,148]. Therefore, a small number of large particles will often mask populations of smaller particles. Accurate bi-modal distributions are impossible unless the size fractions are narrow and over 2-3:1 apart in size (e.g., if size fraction 1 is composed of 10 nm particles, then size fraction 2 must be 20–30 nm to be detected by DLS) [150]. DLS also cannot distinguish between small aggregates and larger particles and cannot reliably measure the PSD of nanoparticles in the presence of plasma proteins [146,149]. Other confounding factors that may distort the data include dust, laser radiation instability, and artificial peaks in polydisperse samples or low-concentration suspensions [151].

DLS is a technique known for providing a PSD that must capture the polydispersity of a nanoparticle dispersion without artificially skewing the distribution’s average [124,148]. Wilson et al. identified that most medicinal nanoparticle products contain a solvated corona, typically composed of polyethylene glycol or other water-soluble polymers [152]. Polymer-stabilized nanoparticles have a surface corona of hydrated polymer chains that contribute to hydrodynamic drag in DLS measurements, and these surface polymers form a collapsed layer in the desiccated, high-vacuum environment of a TEM measurement, leading to what appears to be two different PSDs for the same particle sample. As further discussed by Wilson et al., “TEM size measurements of these particles present two fundamental problems. First, any differential contrast in an unstained sample between the particle core and the comparatively thin corona section can make accurate particle diameter measurement difficult. Second, in conventional TEM, the sample is exposed to high vacuum while on the grid, and the polymer corona collapses into a desiccated layer, which causes particles to appear smaller than the DLS measured hydrodynamic sizes” [152]. They conclude that the PSD obtained from DLS depends on the optimization parameters used to invert the measured autocorrelation function. While they acknowledge DLS can overestimate the PSD standard deviation by a factor of two, the reliability of the average particle size and polydispersity can be assessed by comparison with TEM data. In this case, the TEM measurements should be adjusted to account for the hydrated polymer corona, which influences hydrodynamic size in DLS but appears as a collapsed, desiccated layer in TEM. This analysis highlights that accurate measurement of nanoparticle size distributions and polymer corona thickness, independent of dispersion-based techniques such as DLS, is critical [152]. Additionally, a proper understanding of how the methods work and their limitations is necessary to measure particle size and PSD with confidence and accuracy. For more details on DLS, work by Maguire and coworkers and by Bhattacharjee highlights the principles, parameters, and factors influencing particle size measurement [2,148]. For the reasons above, DLS is considered a low-resolution method [146,147,148,150]. However, despite these faults, we cannot simply abandon DLS as a sizing technique, given its advantages; we should instead consider coupling it with other techniques. By combining DLS with other high-resolution techniques, we can obtain a more complete picture of the PSD for a given sample. For example, asymmetric flow field-flow fractionation (AF4), a separation technique that elutes particles from a polydisperse sample based on size [153], could be coupled with downstream DLS for detection, yielding a clearer PSD and alleviating DLS’s limitations in resolving polydisperse samples [146,154,155,156,157,158,159].

Due to the all-encompassing definition of micromeritics, the measurement of particle properties (size, shape, etc.) and, by default, the techniques by which these measurements were performed, should expand from micron-sized particles to the nano-sized particles used for NEPs. While low-resolution techniques were sufficient when the particle sizes were in the range of tens of micrometers to millimeters (e.g., in the case of the cement industry and formulation of larger pharmaceutical particles such as powders), by the time nanotechnology arrived in the late 1990s, low-resolution techniques were established as applicable and reliable for the measurement of micromeritic properties, but they are not robust enough for characterizing nano-sized particle.

It is not sufficient, however, to just characterize nano-sized particles in their native state. Another requirement for particle characterization should be the analysis of particle behavior in a biological environment, if applicable (e.g., interactions with plasma proteins), which would require a third technique. These interactions often lead to the formation of a protein corona around the particle, even further changing its size and behavior. To examine these effects and elucidate this piece of the picture, a fourth technique would be added. This stacking of characterization techniques, as the complexity of the sample increases, is used to verify the previous method and is known as orthogonality, which is essential in nanomeritics.

## 4. Nanomeritics

Nanomeritics considers particles from 5 nm to 1000 nm (Figure 1). Nanomeritics surpasses the fundamental pharmaceutical micromeritic characterization techniques that focus solely on particle size, distribution, and morphology. Particles rarely come as perfect spheres. This is especially true for nanoparticles, which often assume nonspherical forms such as wires, cubes, and rods, or crystalline powders with a rod-like morphology [160]. While the formulation of micron-sized particles into medicinal products is more established, nanometer-sized formulations are not, and they still fail to reach the market, in part due to a lack of robust, standardized characterization practices [146,161,162]. This review narrows this gap by introducing the concept of nanomeritics, which (1) standardizes the holistic characterization of nanoparticles and (2) defines the key properties that determine stability and biological effects.

At the nanoscale, particle behavior differs markedly from that of their larger counterparts [146]. Chithrani et al. studied how size, coupled with shape, dictates a particle’s interactions with other biological and non-biological surfaces, particles, and molecules [163]. They studied how to vary nanoparticle size and shape to optimize intracellular uptake by using gold nanoparticles of varying sizes (30 nm, 50 nm, 70 nm, and 100 nm) and shapes (circular, rod, and triangular), and measured intracellular gold uptake in HeLa cells. They observed that 50 nm spherical gold nanoparticles exhibited the highest intracellular uptake and hypothesized that their intermediate size and spherical shape were favorable for receptor-mediated endocytosis (RME). Further, they reported that the 50 nm diameter and circular shape contributed to an optimal contact area with cell membrane receptors, thereby maximizing the number of accessible receptor-binding sites for internalization compared with very small or large diameters and rod-like shapes.

One reason promising NEPs fail to reach late-stage clinical trials is insufficient characterization before, during, and after exposure to biological environments (discussed more in Section 5) [11]. Preclinical characterization of nanoparticles is extensive, requiring detailed information on chemical composition, particle size and polydispersity, shape, surface charge, and pharmacokinetic/pharmacodynamic parameters, many of which currently lack standardized methods. If nanoparticle formulations advance to late-stage trials, additional challenges must be overcome, including scale-up issues arising from the small batch sizes used in preclinical studies [11]. The field of nanomeritics seeks to standardize nanoparticle characterization by implementing consistent measurement protocols, using orthogonal techniques such as dynamic light scattering, electron microscopy, and nanoparticle tracking analysis, and employing standardized reporting formats. This approach aims to generate reproducible, comparable data on both physical and biological properties, thereby establishing a comprehensive safety and efficacy profile.

These properties critically influence nanoparticle stability and biological interactions. For instance, when nanoparticles interact with plasma proteins, a protein corona forms around the particle, increasing the hydrodynamic diameter, altering surface charge and hydrophilicity, and potentially masking targeting ligands. These changes can affect cellular uptake, macrophage clearance, and immune activation, ultimately influencing therapeutic efficacy and toxicity (see Section 4.3.1). Compared to micromeritics, nanomeritic characterization integrates additional techniques to examine such interactions, and the use of orthogonal methods ensures reliable results even in complex biological matrices. By standardizing characterization, nanomeritics can reduce batch-to-batch variability, identify stability or immunogenicity risks early, and support rational formulation design. These improvements increase the likelihood that NEPs will succeed in clinical trials and enhance the overall potential of nanomedicines to be safe, effective, and reproducible.

Where nanomeritics first encounters challenges is in the availability of equipment for carrying out orthogonal measurements. DLS is the preferred method due to its ease of operation, affordability of the machines, and relatively high-throughput sample processing. However, as noted, its limited resolution makes it unsuitable as the sole particle sizing technique. Many of the suggested high-resolution methods have caveats. TEM, for example, requires highly trained personnel and a high volume of samples, as exposure to high-energy electrons can destroy the sample. Cryo-TEM, designed to address sample degradation, still requires detailed, time-consuming sample preparation. Additionally, high cost and the need for trained personnel have limited the use of tandem TEM, AF4, and AUC.

While the potential for nanoparticle-based medicines is high, so is the potential of nanomeritics. There are already calls for a better understanding of the methodologies used in nanoparticle assessment, but these are briefly mentioned across several reviews [11,151,164,165,166,167,168]. With the growth of nanomeritics, advances in this field would be available in a central location or via a specific search term, providing easy access for those beginning to characterize a new formulation or those looking to improve nanocharacterization. By evolving into a science rather than just a set of standard operating procedures (SOPs), the future of nanomeritics has implications from industry to academia.

### 4.1. Nanomeritics v. Micromeritics

Nanomeritics is the study and systematic characterization of particles and structures in the size range of approximately 5–1000 nm, extending the principles of pharmaceutical micromeritics to the nanoscale. Pharmaceutical micromeritics focuses on the properties of micron-sized particles, such as size, shape, surface area, charge, mechanical properties, internal microstructure, flowability, and cohesiveness, to evaluate their performance and processing in dosage forms (Figure 5). Metrics such as the Hausner ratio, angle of repose, and Carr’s index provide valuable insights into density and flowability, which are critical for powders and granules in solid formulations. However, these properties are not the primary concerns for nanoparticles or nanosuspensions. At the nanoscale, particle stability and behavior are dominated by molecular forces such as van der Waals and London dispersion interactions, and attributes like particle size, shape, surface area, and charge become the main determinants of formulation performance [169]. Moreover, nanoparticles interact dynamically with biological environments; for example, the adsorption of plasma proteins can lead to the formation of a protein or polymer corona, which alters hydrodynamic size, surface properties, cellular uptake, and ultimately pharmacokinetics and pharmacodynamics. Nanomeritics incorporates integrated measurement strategies to capture both primary attributes, such as size, morphology, surface chemistry, and polydispersity, and secondary attributes that emerge under environmental conditions, including aggregation, degradation kinetics, and corona formation. By systematically characterizing these features across development, manufacturing, and biological contexts, nanomeritics enables a more comprehensive understanding, prediction, and optimization of nanoparticle behavior than is possible using traditional micromeritics alone.

### 4.2. What Defines Nanoscale Particles?

While colloids range in size from 1 to 1000 nm, most nanotechnology typically spans particle sizes of 1 to 100 nm. However, nanotechnology is not just about being small. When particles are created with dimensions of about 1 nm to 100 nm, their properties can change significantly compared to larger particles of the same material, and they can exhibit size-dependent melting temperatures, optical color shifts, and enhanced catalytic activity compared to their bulk counterparts [30].

Pharmaceutical nanotechnology is commonly defined as drug delivery systems with at least one dimension in the nanoscale range (<1000 nm) [27,84,95,170]. Pharmaceutical nanotechnology drug delivery systems can be categorized into three main types: organic, inorganic, and carbon-based nanoparticles. Examples include dendrimers, niosomes, gold and polymeric nanoparticles, nanotubes, quantum dots, liposomes, and polymeric micelles (see Table 1 for a complete classification of nanoparticles). In practice, the smallest drug-delivery nanoparticles are ~5 nm (e.g., gold nanoparticles) [171]. However, the current literature describes pharmaceutical nanotechnology drug delivery systems that are quite large (i.e., nearing 1000 nm) [84,95,170]. It is essential to recognize that nanotechnology extends beyond stand-alone particles. Nanotechnology also influences the properties of much larger structures. For example, nanoemulsions have the same droplet size range as microemulsions but differ in their internal structure and thermodynamic stability [104].

### 4.3. Orthogonality

Nanomeritics employs multiple processes and methods to characterize nanoparticles at each major stage of development. It requires not only the use of orthogonal techniques but also a deeper understanding of the particle behavior itself and those techniques used for analysis. Information on particle size and shape will provide insight into a particle’s behavior, which will allow the quality, safety, and efficacy of a nanoformulation to be assessed. As nanoparticles move from benchtop to a biological space, the need for certainty of proper characterization becomes greater when determining the particle’s clinical future. DLS does not reliably resolve polydisperse samples and cannot, therefore, be relied upon as the sole sizing method. In a literature review conducted over a ten-year period (from 2016 to 2025), 210 primary-source articles that characterized nanoparticle diameter were published in eight journals. Of these, 76.2% used DLS as the sole sizing technique, 11.4% used NTA alone, 10.5% used both DLS and NTA, and only 1.9% used neither method (Figure 6). There was no trend in the use of orthogonal techniques over this period, even though increased use of multiple techniques would have been expected given the well-known limitations of DLS. Since 1995, when Etzler and Sanderson first cautioned about the variability of particle-sizing techniques, there has been a need for multiple confirmatory measurement methods [173]. To address the scrutiny required for preclinical studies and concerns about low-resolution resolving power, an orthogonal approach can be taken. Here, the low-resolution method would be followed by multiple different high-resolution characterization methods. One should follow a low-resolution scanning technique with one or more high-resolution methods and never use a second low-resolution method to confirm a result.

#### 4.3.1. Improving Nanocharacterization

While characterization techniques such as DLS have shortcomings, they are not completely inadequate. DLS is useful for assessing the thickness of the protein corona surrounding nanoparticles. It is important, however, that the pharmaceutical scientist understands the limitations of these techniques and how other high-resolution instruments can complement each other. Measurements of the same nanoparticle may differ, with polymeric nanoparticles often appearing larger when assessed by DLS than by TEM. However, knowledge of each method reveals that during DLS, the particle and its polymer corona remain hydrated, whereas exposure to high vacuums during TEM causes the polymer to collapse into a desiccated layer. For this reason, several recent reviews have called for a standardized protocol or characterization scheme in the nanotechnology arena to improve understanding of the techniques [11,151,167,168]. In one such review by Caputo et al., the joint view of the European Nanomedicine Characterization Laboratory (EUNCL) and the US National Cancer Institute Nanotechnology Characterization Laboratory (NCI-NCL) is the need for more thorough assessments of nanoparticle-enabled medicinal products. The two groups proposed a three-step approach consisting of a pre-screen, a higher-resolution analysis, and, finally, a stringent investigation of NEPs in a biological environment. The first step employs a low-resolution technique, such as batch-mode DLS, to pre-screen a formulation, verify sample integrity, or detect major imperfections. The second step couples two or more high-resolution techniques (particle tracking analysis, asymmetric flow field-flow fractionation, electron microscopy) to resolve the particle size distribution, gain insight into particle morphology, and measure particle concentration. The third step proposed by the EUNCL/NCI-NCL is to assess the critical quality attributes of NEPs under biological conditions. Characterizing nanoparticles before, during, and after their interaction with plasma proteins provides information about the protein corona formed around them and can help elucidate changes in particle size.

Furthermore, the EUNCL offers access to their characterization services, such as assay cascades from physical and chemical characterization (PCC) to in vitro and in vivo biological properties (Table 2), a good first step in educating scientists about the value and necessity of including orthogonal measurements for the characterization of nanoparticles [11]. The NCI-NCL works with the FDA and the National Institute of Standards and Technology (NIST) to develop standardized characterization approaches for physicochemical, pharmacological, toxicological, and immunological use cases. Both agencies’ SOPs for nanocharacterization emphasize the importance of reproducibility through thorough characterization. For example, EUNCL-PCC-01 specifies how to conduct proper size and size distribution measurements using DLS. It recommends that researchers validate the instrument by running a NIST-traceable standard, such as polystyrene latex, and acknowledges conditions that can affect DLS measurements, including absorption, dust contamination, and temperature.

#### 4.3.2. Emerging Technologies

Several newer techniques are extending what can be resolved at the nanoscale. Cryo-electron microscopy (cryo-EM) and cryo-electron tomography image vitrified samples in a near-native, hydrated state, avoiding the drying and staining artifacts that distort soft lipid- and polymer-based particles under conventional EM. They resolve internal features such as lamellarity and cargo distribution and can reconstruct three-dimensional structures for both uniform and heterogeneous particle populations [175]. This detail is valuable for complex formulations such as the lipid nanoparticles used in RNA therapeutics, where bilayer organization and payload location affect stability and efficacy.

Super-resolution fluorescence microscopy, or nanoscopy, resolves features well below the diffraction limit of conventional light microscopy, reaching the 10–20 nm range while retaining the chemical specificity of fluorescence [176]. Single-molecule localization methods and structured illumination microscopy can size individual particles, map surface ligands or cargo, and—using quantitative variants—count the functional groups on a single particle. Because imaging is performed under hydrated, often live-cell conditions, nanoscopy also reveals particle heterogeneity and nano–bio interactions, including cellular uptake and trafficking, which electron microscopy cannot [177]. Its main drawbacks are the need for fluorescent labeling and relatively low throughput.

Machine learning is also being applied to the analysis itself. Convolutional neural networks can segment electron micrographs, separate overlapping particles, and automatically extract size and shape distributions, reducing the labor and operator dependence of manual measurement [178,179]. Other networks sharpen lower-resolution images; one trained on paired optical and electron micrographs of gold nanoparticles reconstructed super-resolution images from blurred optical data [180], while others infer electron-microscopy–grade size and shape parameters from faster, lower-cost measurements such as DLS and UV–visible spectroscopy [181]. Such tools improve throughput, but their output is only as reliable as the reference data on which they are trained.

The lack of translation of nanomedicines from benchtop to bedside is mitigated by a nanoformulation’s successful progression through the in vivo transport cascade and the maintenance of critical quality attributes (CQAs) during scale-up. Overcoming hurdles to biological behavior requires improvements to nano DDS that go beyond passive accumulation in target areas via the enhanced permeability and retention (EPR) effect. Stimuli-responsive strategies have been explored for targeted delivery, including stimuli such as heat, hypoxia, or reactive oxygen species (ROS) [182,183,184]. Further complexities arise when considerations for immune response and the hostile tumor microenvironment (TME), particularly dense stroma and high interstitial fluid pressure, are required. Manipulation of nanoparticle coatings to block macrophage uptake, thereby enhancing immune evasion, aims to extend the delivery system’s lifetime long enough to reach the target site [185,186]. Tunable nanoparticle technology has been developed to extend half-life and evade renal filtration by using a particle size above the glomerular filtration cutoff. This protective shell can then be cleaved by TME-rich enzymes such as matrix metalloproteinases, thereby reducing particle size and allowing deep penetration through dense tumor stroma [187,188].

Further, CQA control is crucial for scale-up from small batches to continuous manufacturing. Hydrodynamic diameter, polydispersity index, and drug loading require real-time monitoring to preserve formulation stability. Pittaway et al. describe the use of inline spatially resolved dynamic light scattering (SRDLS) for real-time particle sizing and PSD during continuous manufacture of poly(*N*,*N*-dimethylacrylamide)-*b*-poly(diacetone acrylamide) (PDMAm-*b*-PDAAm) nanoparticles [189]. AF4-coupled multiple online detectors (MD-AF4) resolve CQAs such as lamellarity (AF4-MALS), encapsulation ratio (AF4-UV/Vis), and colloidal stability/aggregation (AF4-SRDLS), allowing for process parameters, including organic solvent exchange rate, phase mixing ratio, and buffer pH/ionic strength, respectively, to be adjusted accordingly [153,190]. Bian et al. provide a critical review of the multiple configurations for MD-AF4 use in multi-attribute characterization [191]. These strategies can help overcome manufacturing hurdles and improve the nanomedicine scale-up.

### 4.4. Nanomeritic Effects Within Biological Environments

#### 4.4.1. Particle and Protein Coronas

During nanoparticle formation, a particle corona, or spontaneously formed coating, develops around the particle. Johnson et al. demonstrated the mechanism of formation from the rapid assembly of block copolymers [192]. Supersaturated copolymer concentrations and a homogeneous solution led to rapid nanoparticle nucleation. Upon fusion of polymer unimers, a brush layer forms as the particle reaches a critical size. This brush layer, or brush corona, serves as the rate-limiting barrier through which free polymer must form new particles or fuse with existing particles, and it has a low insertion barrier at the brush corona. Via this diffusion-limited mechanism, the formed particles become kinetically frozen, with little exchange of solvent or unimers with the particles.

The particle corona has also been identified as a major contributor to the particle’s free energy. Core chain stretching, corona chain repulsion, and the core/corona interfacial tension are three governing principles of block copolymer micelles [95,193]. While block copolymer molecular weight, block ratio, and concentration have been shown to influence several micelle attributes, such as morphology and critical micelle concentration, the solvent composition can also affect micelle size and shape [193,194,195,196]. Cooksey et al. demonstrated the tunability of block copolymer micelles by varying the solvent composition from 10 to 60% deuterated water/tetrahydrofuran [197,198]. Increases in tetrahydrofuran in the bulk solvent led to increased solvent uptake into the micelle core and into the lower molecular weight micelle as a result of changes in the core–corona interfacial tension. Durgun and colleagues also explored the tuning of particle size of fluorescent silica nanoparticles by controlling the amounts of ethanol and ammonia during synthesis, with increased ethanol yielding particles with a smaller hydrodynamic diameter and increased ammonia yielding particles with a larger hydrodynamic diameter [198]. Extensive work has been done to show the relationships among solvent composition, particle size, and core–corona formation. Mixing induces a rapid change in solvent composition, leading to dynamic aggregation of block copolymers as the solubility of the hydrophobic block decreases. The aggregates grow and mature into kinetically trapped (frozen) nanoparticles with a hydrophobic core and a hydrophilic corona. The nanoparticles become frozen because the low solubility of individual block copolymer chains in the final mixture prevents further growth, and the hydrophilic corona inhibits nanoparticle fusion. Solvents with high water affinity and low viscosity enhanced solvent diffusion into the aqueous phase, thereby forming smaller nanoparticles due to the rapid change in solvent quality, χ. For solid lipid nanoparticles, the size increased with solvent viscosity [199,200].

The micelle size and aggregation number are also impacted by the composition of the solvent mixture. An increase in the concentration of the selective solvent, and the associated increase in interfacial tension, generally increases the aggregation number (and reduces the number of micelles while increasing the micelle core radius), thereby minimizing the total core−corona interfacial area of the overall micellar system [1,27,200,201].

When the particles are dispersed in an aqueous medium, an electrical corona or electrical double layer forms. Here the electric double layer consists of three parts: (1) particle surface charge—charged ions (commonly negative) adsorbed on the particle surface; (2) Stern layer—counterions (charged opposite to the surface charge), attracted to the particle surface and closely attached to it by the electrostatic force; and (3) diffuse layer—a film of the dispersion medium. The diffuse layer contains free ions with a higher concentration of the counterions. The zeta potential is the electrical potential at the slipping plane.

It is important to understand the interactions between particles and plasma proteins, as bound plasma proteins alter the biological properties of nanoparticles [11,202,203,204,205,206,207]. The results of nanoparticle–plasma protein interactions can range from modest changes in particle size due to the formation of a protein corona (i.e., a layer of plasma proteins covering the entire surface of the nanoparticle, thus increasing the overall size) to large increases in particle size due to aggregation with other particles. The changes in corona size across different media are shown in Figure 7. Plasma protein–nanoparticle interactions do not just lead to particle size increases. Disaggregation and destabilization of nanoparticles are also known to occur after interactions with plasma proteins [202]. Sizing analysis in complex media requires high-resolution techniques because of the complexity of the nanoparticle–protein mixture. For example, if a nanoparticle is injected into the bloodstream, it will encounter various plasma proteins, such as serum albumin, where a protein corona will form [203,204]. Over 125 different plasma proteins have been detected on nanoparticle surfaces [205]. The first layer (i.e., the Stern layer) is a hard corona, which is tightly bound and stable. The second (i.e., diffuse) layer, called the soft corona, is loosely attached to the hard corona and is composed of substances with various charges and sizes. This hard corona forms as abundant low-affinity proteins of the soft corona are replaced by higher-affinity proteins [206]. It is important to note that the protein corona and nanoparticle complex remain intact during cellular binding, internalization, and transport [207]. The binding of serum proteins affects not only nanoparticle pharmacokinetics but also their immunogenicity.

#### 4.4.2. In Vivo Behavior and Pharmacokinetics

Nanometric properties, including size, shape, and surface chemistry, affect the pharmacokinetics of nanoparticles in vivo. Size, corona structure, and surface chemistry of nanoparticles affect the mechanisms by which nanoparticles can cross primary and secondary barriers and reach their sites of action [208]. Macedo et al. studied pharmacokinetic data collected from nanoparticle delivery in multiple tumor-bearing mice and observed that nanoparticles in the size range of 10–100 nm were significantly higher in area under the curve (AUC) and lower in volume of distribution (V_d_) and total clearance (CL) than nanoparticles less than 10 nm [209]. The smallest nanoparticles (<10 nm) are filtered by the kidneys and are thus easily cleared. They also observed that zeta potential, particularly neutrally charged nanoparticles, again exhibit higher AUC and lower V_d_ and CL than positively charged nanoparticles, likely due to less interaction with charged biological entities such as cell membranes.

Furthermore, the nanoparticle material influences their pharmacokinetic parameters. Macedo et al. observed that liposomes had the highest AUC and the lowest V_d_ and CL compared to gel, micelles, and gold nanoparticles [209]. Furthermore, Kumar et al. highlight how material composition influences nanoparticle pharmacokinetics, as reflected by nanoparticle biodistribution coefficients (NBCs) [210]. Lipid-based nanoparticles showed the greatest circulation stability, with NBCs of 16.4% ID of nanoparticle per gram of tissue (%ID/g) in blood and 16.2%ID/g in the kidney, indicating increased systemic exposure and renal clearance. Polymeric nanoparticles performed similarly, with blood levels of 13.8%ID/g and particularly high spleen uptake (12.6%ID/g), suggestive of longer circulation and recognition by the reticuloendothelial system (RES). However, inorganic nanoparticles, including gold, iron oxide, and silica, were distributed differently. Gold nanoparticles accumulated in the liver at 13.6%ID/g and exhibited broad tissue distribution overall. Iron oxide nanoparticles demonstrated more distribution in the spleen (10.2%ID/g), consistent with their recognition by macrophage-rich tissues, while silica nanoparticles showed delayed distribution in the heart, achieving 3.7%ID/g after 24 h. Both authors remark that a combination of nanoparticle properties, such as size and material, affects their overall pharmacokinetic profiles. The study of nanomeritics, which provides the holistic characterization of nanoparticle properties, provides the necessary approach to studying the pharmacokinetics of potential nanoparticle therapeutics. Building a thorough NEP profile (size, morphology, surface coatings, and charges) gives an opportunity to bridge their promising preclinical performance with in vivo implementation: a gap in which most products fail that is further widened by regulatory barriers.

## 5. Regulatory Hurdles

Regulatory agencies such as the US Food and Drug Administration (FDA), the European Medicines Agency (EMA), and Japan’s Pharmaceuticals and Medical Devices Agency (PMDA) have introduced regulatory challenges that have slowed the translation of nanomedicine. Regulatory agencies have increasingly made explicit their expectations for how nanomedicines should be characterized. In the United States, the FDA finalized guidance for industry in 2022, “Drug Products, Including Biological Products, That Contain Nanomaterials”, which adopts a risk-based framework and asks sponsors to characterize attributes such as chemical composition, average particle size, and size distribution, morphology, surface properties, including charge and coating, and physical and chemical stability [211]. A central theme of the guidance is not characterization for its own sake but establishing how these attributes relate to the quality, safety, and efficacy of the finished product, and it encourages the use of complementary, fit-for-purpose methods alongside recognized standards where they exist [211,212].

The EMA has taken a complementary, more product-specific approach. Beginning in 2011, its Committee for Medicinal Products for Human Use issued a series of reflection papers on the characterization and data requirements for particular classes of nanomedicine, including surface-coated nanoparticles, intravenous liposomal products, and iron-based nanocolloidal preparations [213,214]. These papers emphasize the physicochemical attributes that govern in vivo behavior, such as coating stability, particle size, and surface properties, as well as the comparability data required when a follow-on nanosimilar is developed against an innovator product [215]. Even so, neither agency yet operates with fully harmonized definitions or standardized reference methods, and systematic efforts to map existing measurement standards against regulatory needs have revealed substantial gaps [212,216]. The practical implications reinforce the orthogonal approach advocated here: regulators expect nanoparticle properties to be established by multiple validated methods and tied explicitly to clinical performance.

There are several regulatory hurdles that nanomedicines face as compared to small-molecule drugs. Since many nanomedicines span traditional boundaries among drugs, devices, and biologics, an unintentional bottleneck has arisen due to overlapping regulatory jurisdictions within a single agency [217,218,219]. Neither the FDA nor the EMA has established a distinct, stand-alone legal category for nanomaterials, partly due to differing regional definitions, which complicate global development efforts [161,211,217,218,219]. Instead, nanomedicines are reviewed under traditional biological or small-molecule frameworks. Furthermore, divergent definitions across regions require developers to conduct redundant analytical and preclinical trials to satisfy the various global authorities. Generic nanomedicines also face significant challenges (e.g., the need to demonstrate identical chemical structures) [220,221,222]. However, for complex nanomedicines, an identical physical structure cannot be fully proven using standard methods.

Furthermore, the additional regulatory requirements (e.g., detailed tissue distribution, organ accumulation, etc.) for assays beyond blood concentration (standard pharmacokinetics) have significantly delayed the market entry of generic nanomedicines. Due to the complexity of nanomedicines, characterization requirements have increased dramatically. Single-method characterization is not a viable option for nanomedicines. Rather, multi-technique orthogonal testing (e.g., DLS paired with AF4-MALS, Cryo-TEM, and NTA) is standardly required. This requirement provides independent, non-redundant verification of particle size distribution, aggregation state, concentration, and morphology across production lots [146,191,223,224]. Also, the evaluation of nanomedicines in complex biological fluids to assess the protein corona, batch-to-batch changes in surface charge, and aggregation risks is routinely required. To address these regulatory challenges, there is a great need for harmonized definitions, standardized characterization protocols, and clear regulatory pathways for nanomedicines, thereby facilitating faster market access and innovation.

## 6. Discussion and Future Directions

The science of small particles, known as micromeritics, laid the foundation for future work on particle characterization. Resolving particle size distributions, studying shape, and gaining insight into charge are all key micromeritic evaluations necessary for determining the function and behavior of a particle. DLS, which has established itself as the primary sizing technique due to its rapid, inexpensive analysis, is not without its faults; while it may suffice for larger particles, it lacks the resolving power for smaller nano-sized particles. Supplementing DLS with other high-resolution techniques, such as sorting through AF4 prior to size analysis or the use of multiple additional confirmatory methods, such as EM, particle tracking analysis (PTA), or online multi-angle light scattering (MALS), is the basis of many multi-step characterization approaches and an important pillar of nanomeritics. With the continued expansion of nanotechnology, we propose the term nanomeritics to highlight the unmet need for stronger, standardized characterization of nanoparticles that extends far beyond micromeritics. Beyond that, we intend for nanomeritics to function as the science of nanoparticles, from understanding how particle size dictates their fate in biological environments to recognizing that molecular forces are critical to the stability of nanoformulations. The future of nanomeritics is bright and represents the first step toward answering complex questions about the fate of NEPs. The most recent approved nanoparticle medicinal product is Onpattro, approved in 2018. Onpattro is used to treat polyneuropathy in patients with hereditary transthyretin-mediated amyloidosis. Onpattro’s formulation consists of a small interfering RNA (siRNA) containing a PEGylated lipid nanoparticle. The lipid nanoparticle protects the active siRNA, ensuring its delivery to the liver. The careful characterization of the formulation itself led to successful approval, which remains rare to date. With more comprehensive guidelines for nanocharacterization methods, we predict that more NEPs will be approved more quickly [225,226].

These scientific and regulatory uncertainties ultimately translate into financial risk. The expense of scale-up, the breadth of required safety and immunotoxicity testing, and the length and unpredictability of regulatory review can together discourage the development of an otherwise promising candidate [225]. Expanding the clinical nanomedicine pipeline will therefore depend not only on improved characterization but on more robust manufacturing, more predictive biological models, and a more coherent regulatory framework.

In summary, nanomeritics strategies, including orthogonal particle characterization techniques and enhanced nanoparticle–biological interface interactions, will enable significant strides in the translation of nanoparticles into therapeutics. Armed with this knowledge from fundamental micromeritics, nanomeritics is well-positioned to expand and improve the discovery, development, and efficacy of future NEPs.

## Figures and Tables

**Figure 1:**
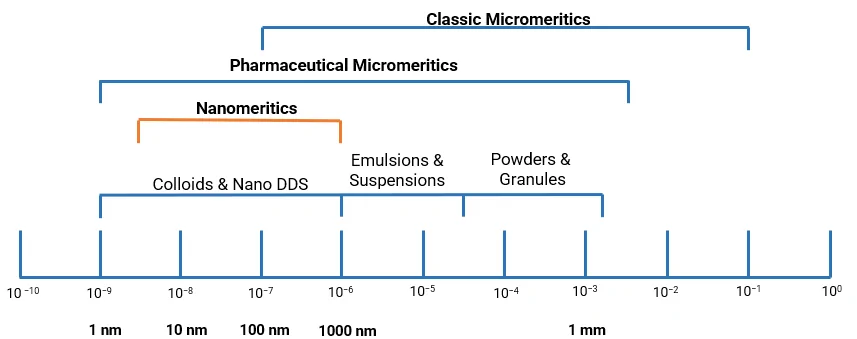
Size ranges of classic micromeritics, pharmaceutical micromeritics, and nanomeritics. DallaValle introduced classic micromeritics, covering a size range from 100 nm to 100 mm. As particles for pharmaceutical applications emerged, the term pharmaceutical micromeritics was coined and expanded to pharmaceutical particles ranging from 1 nm to a few millimeters [26]. From smallest to largest, pharmaceutical micromeritics includes colloids and nano drug delivery systems, emulsions and suspensions, and powders and granules. Today, the development of nanoscale particles for pharmaceutical applications has surged. We designate the term nanomeritics to describe the study of particles in the size range of 5–1000 nm for pharmaceutical applications.

**Figure 2:**
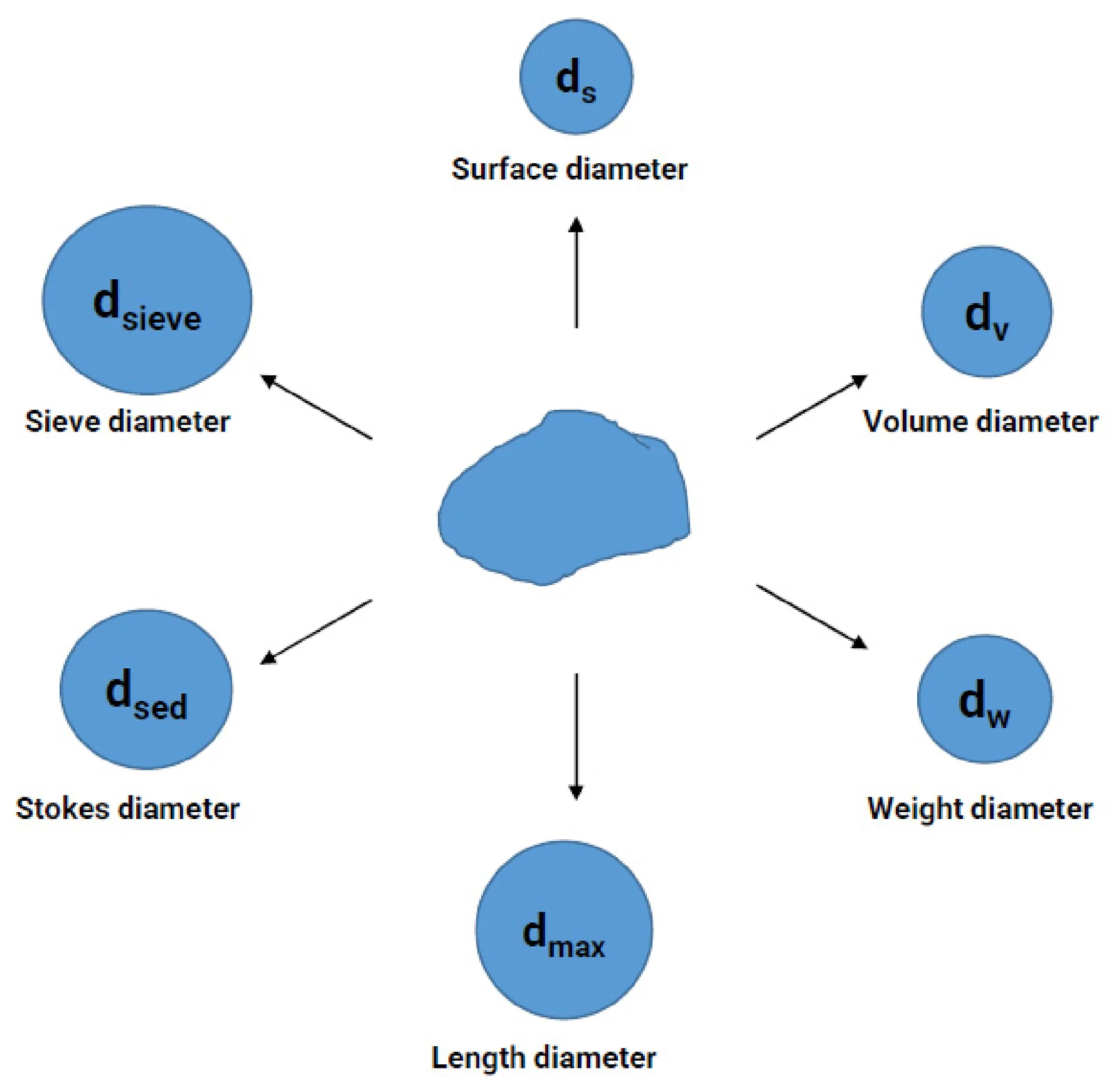
Equivalent spheres for the characterization of imperfect particles in pharmaceutical sciences. The concept of equivalent spheres simplifies the measurement of imperfect particles. A sphere with a diameter that has the same surface area, volume, weight, length, or sedimentation rate is related to the asymmetric particle being measured. Equivalent spheres have advanced particle characterization techniques by delineating the type of diameter measured. (Re-drawn and modified from ref [Worldwide, M. I. A Basic guide to particle characterization. 1–24 (2015)] [54]).

**Figure 3:**
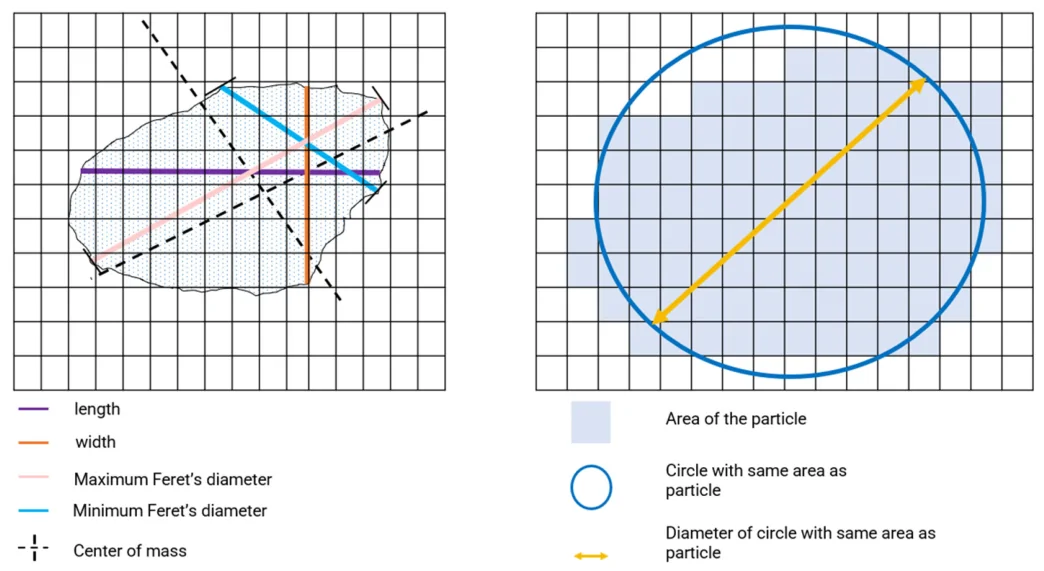
Particle shape analysis. Image analysis converts complex three-dimensional particles into a two-dimensional projection to measure particle shape. Parameters used include Feret diameters, length and width, and the center of mass. Feret diameters are the longest (maximum) and shortest (minimum) distance between any two parallel tangents on the particle (**left**). Imaging software produces a two-dimensional projection of the particle, from which particle shape parameters are calculated using direct measurements of the diameter, perimeter, and other linear parameters from the projected pixel image (**right**). An equivalent sphere having the same area as the projection can be used to estimate size. (Re-drawn and modified from Figure 19-5 of Martin, Pharmaceutical Micromeritics. 19-5 (2023) [26]).

**Figure 5:**
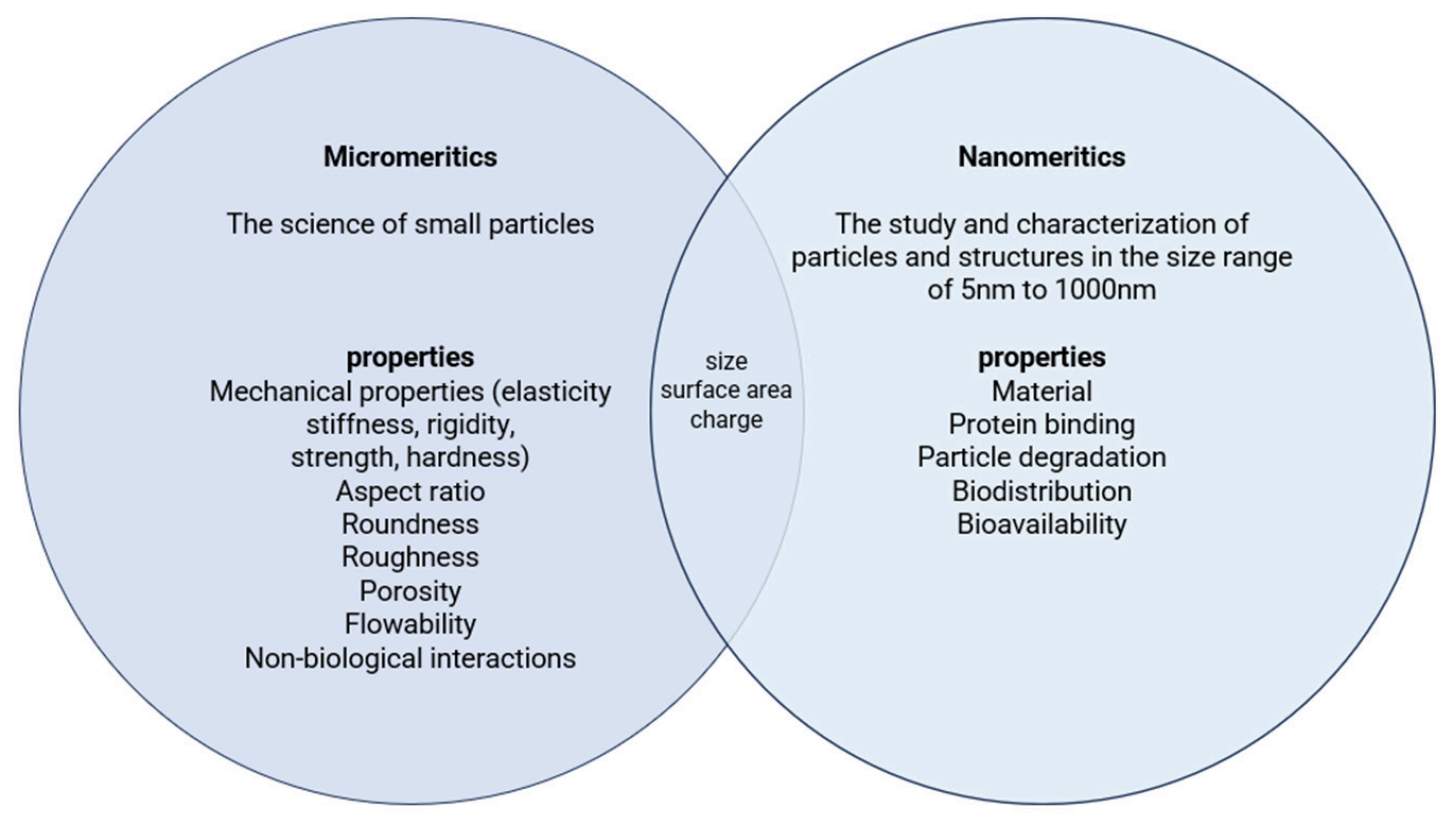
Comparing micromeritics and nanomeritics. Micromeritics is the science of small particles, whereas nanomeritics is the study and characterization of particles and structures in the size range of 5–1000 nm, with a focus on biological properties. Properties such as protein binding, particle degradation, biodistribution, and bioavailability significantly affect the performance and efficacy of nanoparticle-enabled medicinal products (NEPs).

**Figure 6:**
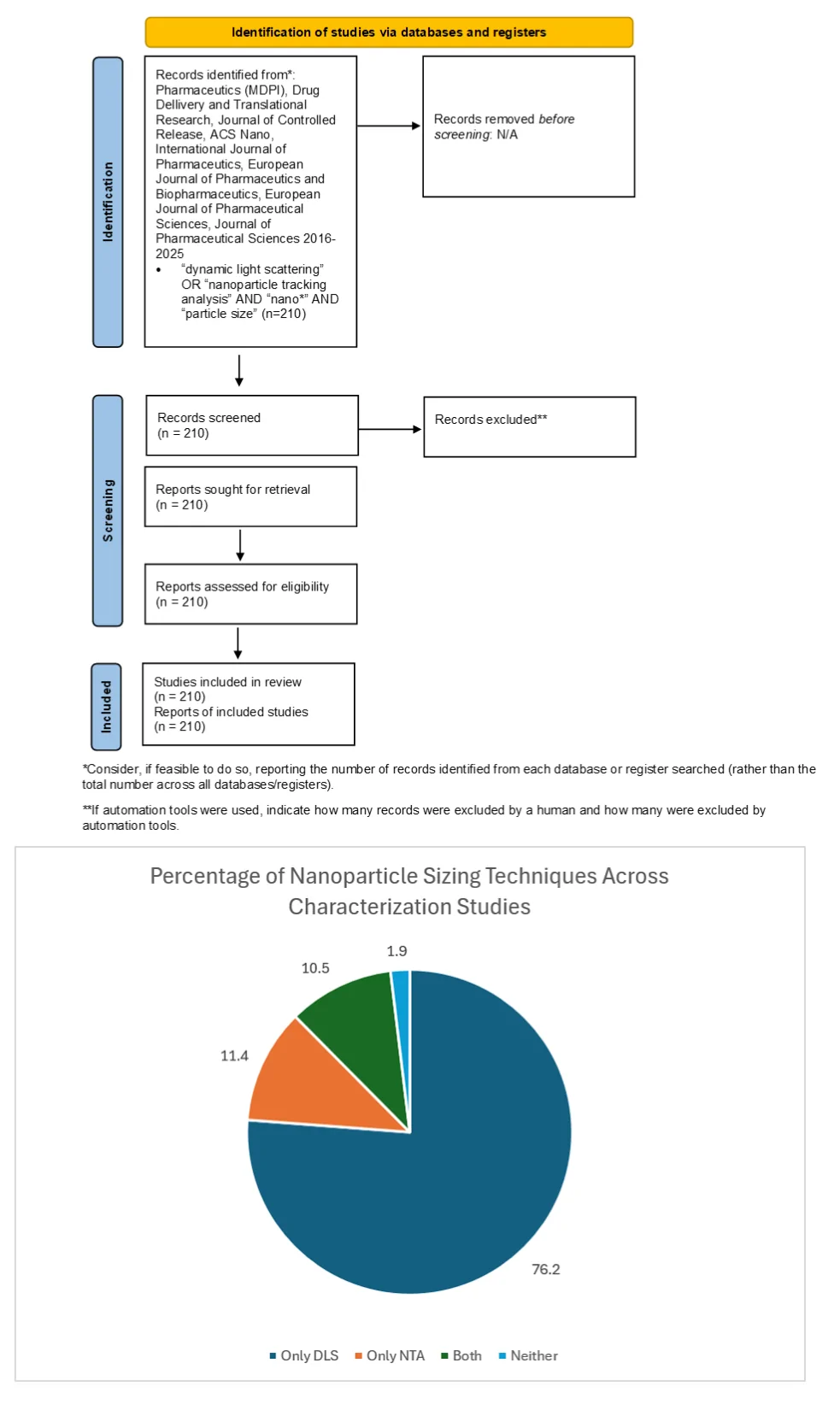
Literature review on sizing techniques utilized across characterization studies. (**upper**) Summary of literature search strategy. (**lower**) Percentage of nanocharacterization studies published from 2016 to 2025 across eight journals utilizing dynamic light scattering, nanoparticle tracking analysis (NTA), both, or neither technique. N = 210.

**Figure 7:**
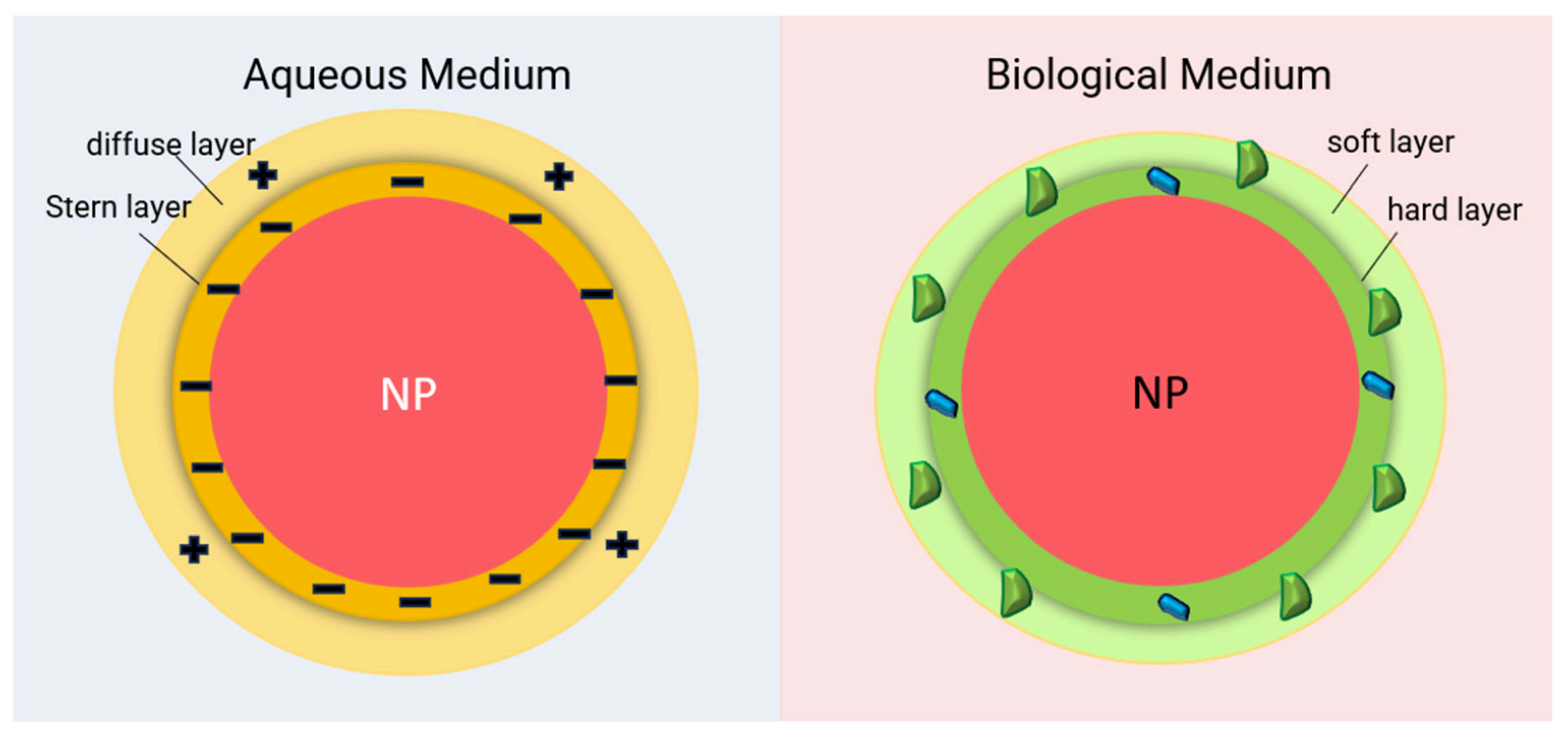
Formation of a corona in an aqueous medium and a biological medium. The corona is a spontaneously forming coating that develops around the particle. In an aqueous medium, an electrical double layer forms, consisting of three parts: charged ions (typically negative) adsorbed at the particle surface, a Stern layer containing counterions (oppositely charged to the surface charge), and a diffuse layer that serves as a film in the dispersion medium. In a biological medium, the first layer is hard, tightly bound, and stable. The second layer, called the soft layer, is loosely attached to the hard corona and consists of particles with varying charges and sizes.

**Table 1:** Nanoparticle categorization. Classification of nanoparticles based on their composition, dissolvability, and size range gathered from the literature. Nanoparticles are classified as either organic, inorganic, or carbon-based. Most nanoparticles are found in the 1–100 nm range, but some extend into the 1000 nm range.

Category	Subcategory	Size Range (nm)	Notable Examples	Reference
Organic	Carbohydrates	1–100	Carbohydrate based dendrimers—sugar core with branched carbohydrate chains	[30]
	Lipids	Lipids 10–1000 Liposomes 20–1000	Liposomes—spherical shaped vesicles with 1+ phospholipid bilayers	[30,172]
	Polymeric	Polymeric nanoparticles (NPs) 1–1000	PCL, PLA, PLGA nanospheres	[13,30]
	Protein Bound	Albumin bound NPs such as Abraxane are ~130	Albumin NPs (e.g., Abraxane—NP albumin bound formulation of paclitaxel)	[30]
Inorganic	Metals	AgNPs: 1–100 CuNPs: 1–100	Cu, Ag, Au NPs	[14,30]
	Ceramic (carbonates, phosphates, oxides of metals ex. Ti or Ca)	TiO_2_: 3–10 SiO_2_: 7	Titanium oxide (TiO_2_), Alumina (Al_2_O_3_), silica (SiO_2_)	[14,30]
	Semiconductors (metal/nonmetal that absorbs/emits light)	1–100	ZnO NPs	[30]
Carbon Based	N/A	<1	Fullerenes—closed carbon cage structure	[30]
		10–500	Carbon black NPs—grape like/spherical aggregates	[30]

**Table 2:** Categorization of assays for each characterization step of nanoparticles. Assays can be categorized into pre-screening, physicochemical characterization, in vitro immunological, or in vitro hematological studies. The European Nanomedicine Characterization Laboratory (EUNCL) identifier column links to the relevant assay protocol on the EUNCL website. The protocol provides general guidelines and recommendations for the specific nanocharacterization assay.

Characterization Step	Assay	EUNCL Identifier	Reference
Pre-Screening	Size, size distribution	EUNCL-PCC-01	[174]
	Zeta potential	EUNCL-PCC-02	
	pH of NP suspension	EUNCL-PCC-13	
	Endotoxin by kinetic turbidity assay	EUNCL-STE-01.2, 01.3, 01.4	
	Detection of mycoplasma	EUNCL-STE-03	
	Microbial contamination	EUNCL-STE-02.2	
	Drug loading	EUNCL-PCC-30	
	Detection of Gram-negative endotoxin	EUNCL-STE-001.2.1	
Physiochemical Characterization	Cryo-TEM	EUNCL-PCC-20	
	Measuring NP aggregation propensities with DLS	EUNCL-PCC-21	
	Size, Size Distribution	EUNCL-PCC-22	
	Free vs. encapsulated drug	EUNCL-PCC-31	
	Lipid Composition	EUNCL-PCC-32	
	Polymer Composition	EUNCL-PCC-33	
	Drug Release in Complex Media	EUNCL-PCC-035	
	Particle Tracking Analysis	EUNCL-PCC-023	
In Vitro Immunology	Hematolytic properties	EUNCL-ITA-01	
	Platelet Aggregation	EUNCL-ITA-02.1, 02.2	
	Complement Activation by EIA	EUNCL-ITA-005.2	
	Plasma Coagulation Time	EUNCL-ITA-12	
	Plasma Protein Interactions	EUNCL-ITA-04	
In Vitro Hematology	Leukocyte proliferation	EUNCL-ITA-006.2	
	Induced Inflammatory Cytokines	EUNCL-ITA-10	

## Data Availability

Dataset available on request from the authors.
